# Characteristics of Seed Vigor in Rice Varieties with Different Globulin Accumulations

**DOI:** 10.3390/ijms23179717

**Published:** 2022-08-26

**Authors:** Liling Peng, Hulun Lu, Jiajin Chen, Ziyan Wu, Zitong Xiao, Xindong Qing, Jintao Song, Zhoufei Wang, Jia Zhao

**Affiliations:** The Laboratory of Seed Science and Technology, Guangdong Key Laboratory of Plant Molecular Breeding, South China Agricultural University, Guangzhou 510642, China

**Keywords:** rice, seed vigor, globulin protein, reactive oxygen species, direct seeding

## Abstract

Seed vigor of rice is an important trait for direct seeding. The objective of this study was to reveal the relationship between globulin and seed vigor, and then to explore a method for evaluating seed vigor. Several rice varieties with different levels of 52 kDa globulin accumulation were used to compare seed vigor under normal and aged conditions. Results showed that varieties with high globulin accumulation obtained significantly higher seed vigor, measured by germination percentage and germination index, compared with those varieties with low globulin accumulation under normal and aged conditions. Meanwhile, a significantly higher accumulation of reactive oxygen species (ROS) was observed in the early germinating seeds of varieties with high globulin accumulation compared to those varieties with low globulin accumulation under normal and aged conditions. Collectively, the globulin content could be applied in the evaluation of seed vigor, which contributes to the selection of rice varieties for direct seeding.

## 1. Introduction

Rice (*Oryza sativa* L.) is the most widely cultivated cereal crop in China. Recently, the approach of direct seeding has been widely applied because of its cost- and labor-saving advantages [1]. Seed vigor is an important trait that influences rapid seed germination, good seedling growth, and better aging tolerance, which is a crucial factor in determining field performance, and crop yield [2,3,4]. Thus, the selection of varieties with high seed vigor is important for direct seeding of rice.

Seed germination is a crucial event for the transition from seed to seedling in rice. The mobilization of stored reserves such as carbohydrate, protein, and lipid play a vital role during seed germination [5]. For example, seed storage proteins (SSPs) provide free amino acids to the embryonic axis and energy for seed germination [6,7]. In rice, seed storage proteins are composed of glutelin, albumin, globulin, and prolamin. It has been reported that the lack of globulin synthesis regulates the accumulation of seed storage proteins during seed development in rice [8]. Recently, we observed that the cupin domain containing protein gene *OsCDP3.10* regulates the accumulation of 52 kDa globulin, which is involved in the regulation of seed vigor by influencing amino acid and hydrogen peroxide (H_2_O_2_) levels [9]. However, the characteristics of seed vigor in rice varieties with different globulin accumulation are still unclear.

Reactive oxygen species (ROS), such as superoxide anion (O_2_^−^) and hydrogen peroxide (H_2_O_2_), are highly reactive molecules [10]. It has been reported that ROS are involved in the regulation of seed dormancy, seed germination, and seed aging tolerance [11,12]. For example, seed dormancy can be released by low levels of ROS as signaling particles, triggering seed germination [12,13]. However, it is well known that high levels of ROS will cause seed deterioration [11], which will influence seed viability and seed quality during storage. Due to the abundance of SSPs in seeds and their high affinity for oxidation, SSPs are a primary target for oxidation [14]. In *Arabidopsis*, the 12S globulin genes have been reported to have involvement in the regulation of seed aging tolerance [15]. However, the relationship between seed aging tolerance and globulin accumulation are poorly understood in rice.

To reveal the relationship between seed vigor and globulin accumulation, and to explore methods for evaluating seed vigor, rice varieties with different globulin accumulation were used to investigate the characteristics of seed vigor in this study. We observed that rice varieties with high globulin levels possessed better seed vigor, including of speed germination, and seed aging tolerance. Globulin accumulation contributing to seed germination might be through accumulation of ROS that acts as signals during early seed germination. Thus, globulin content is a potential trait for the selection of rice varieties with high seed vigor for direct seeding in rice.

## 2. Results

### 2.1. Variation of Globulin Accumulation in Rice

**Figure 1:**
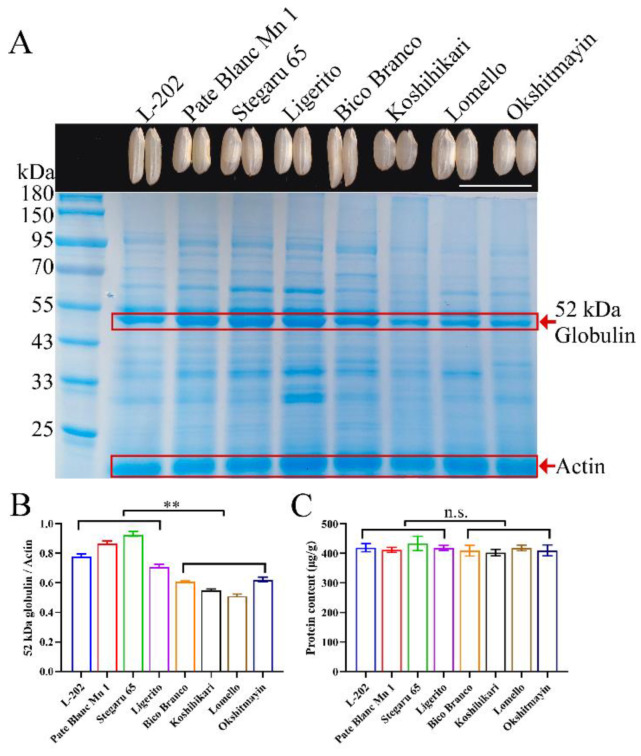
Comparison of protein content among varieties. (**A**) 52 kDa globulin accumulation. (**B**) The quantified 52 kDa globulin accumulation by using ImageJ. (**C**) Total protein content. Each column represents the means ± SD, *n* = 3. ** indicates the significant difference at the 1% level according to Student’s *t*-test. n.s. represents not significant.

To reveal the variation of globulin accumulation in rice grains, a total of 40 varieties were used to detect the 52 kDa globulin content. SDS-PAGE analysis showed that there were significant differences in 52 kDa globulin accumulation among varieties. To further investigate the roles of globulin on the total protein content, four accessions (L-202, Pate Blanc Mn 1, Stegaru 65, and Ligerito) with high globulin content and four varieties (Bico Branco, Koshihikari, Lomello, and Okshitmayin) with low globulin content were randomly selected for the evaluation of total protein content (Figure 1A,B). Interestingly, there was no significant difference in the total protein content among these varieties (Figure 1C). Thus, these eight varieties were used to further investigate the relationship between globulin content and seed vigor.

### 2.2. Globulin Content Involves in Seed Vigor

**Figure 2:**
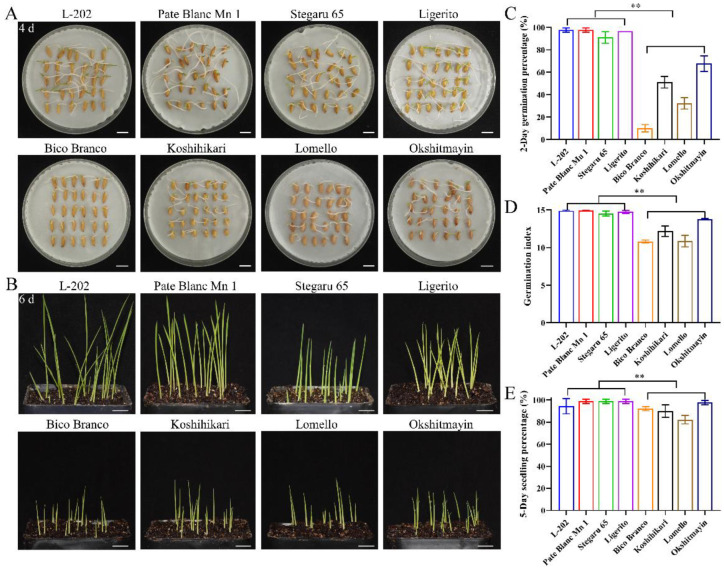
Comparison of seed vigor among varieties under normal conditions. (**A**) Representative images of the seed germination in Petri dishes. (**B**) Images of seedling establishment from direct seeding in soils. Bar = 10 mm. (**C**) Two-day germination percentage; (**D**) Germination index; (**E**) Five-day seedling percentage. Each column represents the means ± SD, *n* = 3. ** indicates a significant difference at the 1% level according to Student’s *t*-test.

**Figure 3:**
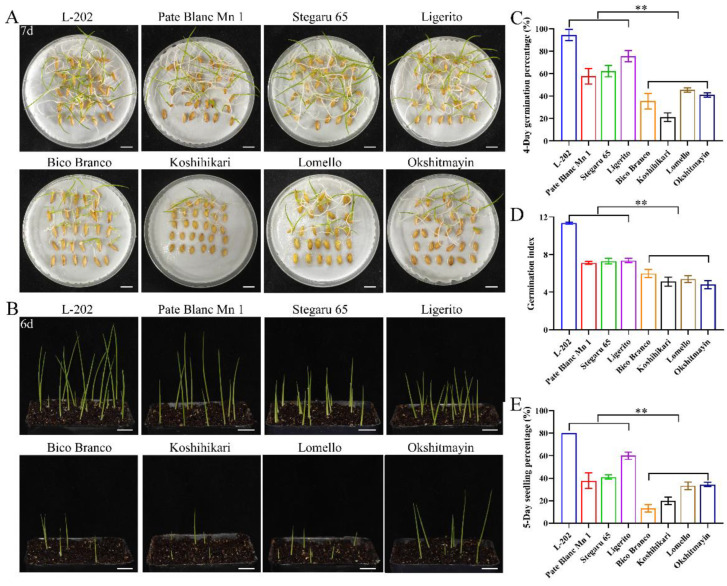
Comparison of seed vigor among varieties under aged conditions. (**A**) Representative images of the seed germination in Petri dishes. (**B**) Images of the seedling establishment in direct seeding in soils. Bar = 10 mm. (**C**) Four-day germination percentage; (**D**) Germination index; (**E**) Five-day seedling percentage. Each column represents the means ± SD, *n* = 3. ** indicates a significant difference at the 1% level according to Student’s *t*-test.

The seed vigor of the aforementioned eight varieties of rice was identified under normal and aged conditions. Significantly higher seed vigor was observed in varieties (L-202, Pate Blanc Mn 1, Stegaru 65, and Ligerito) with high 52 kDa globulin accumulation compared with those varieties (Bico Branco, Koshihikari, Lomello, and Okshitmayin) with low 52 kDa globulin accumulation under both conditions (Figure 2 and Figure 3). The traits of germination speed, including germination percentage and germination index, were significantly higher in varieties with high globulin accumulation (Figure 2A,C,D and Figure 3A,C,D). Meanwhile, good seedling growth was observed in varieties with high globulin accumulation in direct seeding in soils (Figure 2B,E and Figure 3B,E). The mean relative germination percentage, germination index, and seedling percentage of varieties with high globulin accumulation as compared to the varieties with low globulin accumulation were 2.35, 1.24, and 1.08 under normal conditions, respectively, while the corresponding data were 2.02, 1.55, and 2.16 under aged conditions. These results suggest that greater accumulation of globulin contributes to seed vigor, including germination speed, seedling growth, and seed aging tolerance, in rice.

### 2.3. Globulin Affects Reactive Oxygen Species during Seed Germination

**Figure 4:**
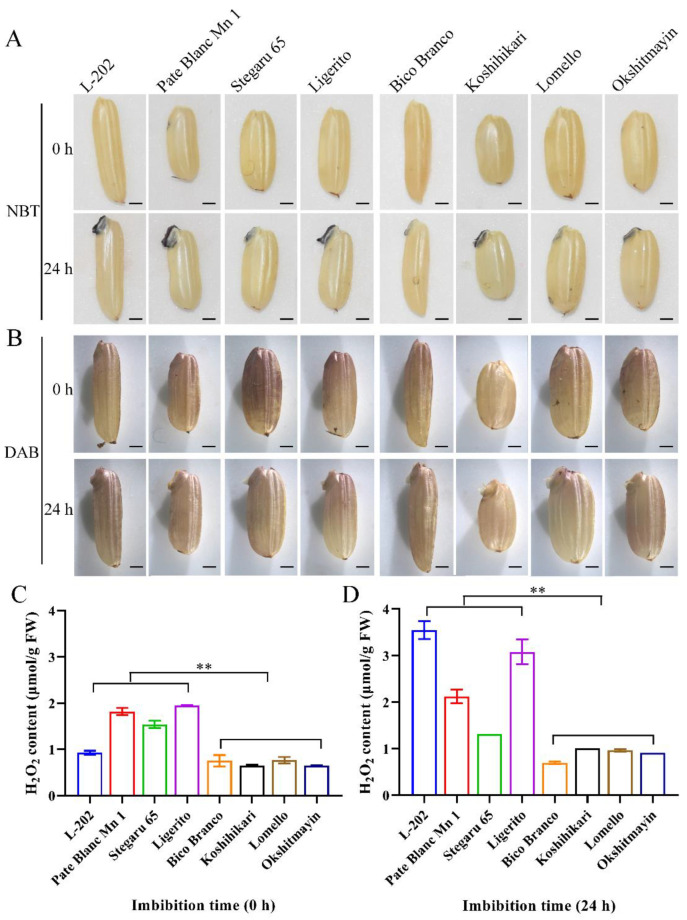
Comparison of ROS levels among varieties during seed germination under normal conditions. (**A**) NBT staining analyzed the O_2_^−^ accumulation; (**B**) DAB staining analyzed the H_2_O_2_ accumulation; (**C**) H_2_O_2_ content of dry seed; (**D**) H_2_O_2_ content of imbibition seeds (24 h) at 25 ± 1 °C in the darkness. Bar = 1 mm. Each column represents the means ± SD, *n* = 3. ** indicates the significant difference at the 1% level according to Student’s *t*-test.

**Figure 5:**
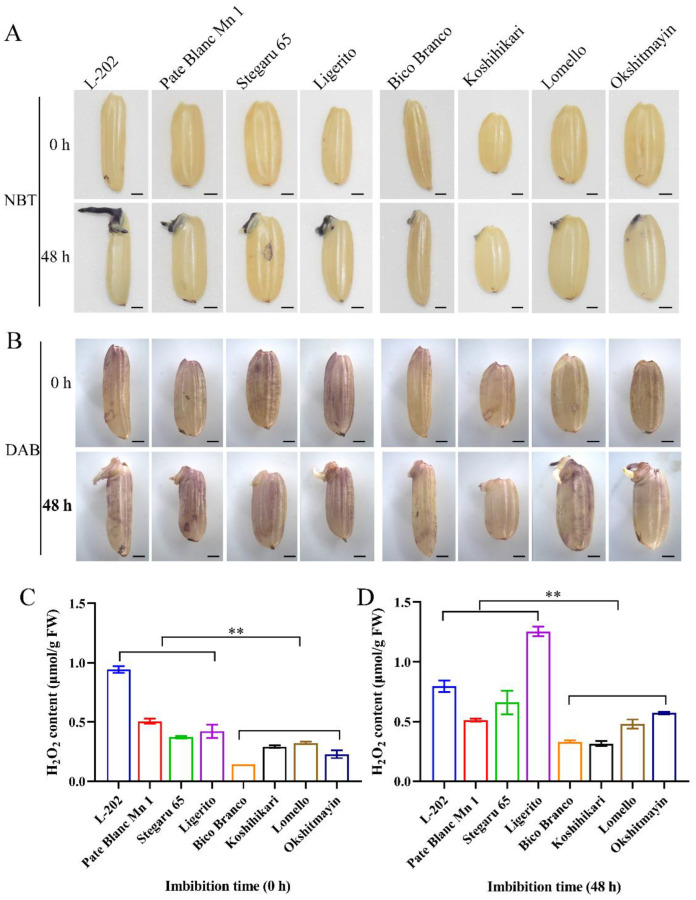
Comparison of the ROS level among varieties during seed germination under aged conditions. (**A**) NBT staining analyzed the O_2_^−^ accumulation; (**B**) DAB staining analyzed the H_2_O_2_ accumulation; (**C**) H_2_O_2_ content of dry seed; (**D**) H_2_O_2_ content of imbibition seeds (48 h) at 25 ± 1 °C in the darkness. Bar = 1 mm. Each column represents the means ± SD, *n* = 3. ** indicates a significant difference at the 1% level according to Student’s *t*-test.

Previous studies have revealed that the 52 kDa globulin gene *OsCDP3.10* influences seed vigor by regulating reactive oxygen species (H_2_O_2_) during early germination [9]. To further confirm whether globulin affects seed vigor through the ROS pathway, a comparison of H_2_O_2_ and O_2_^−^ accumulation was conducted in dry and germinating (24 h imbibition) seeds using NBT and DAB staining methods under normal and aged conditions. A reddish-brown was observed in the embryo and testa of varieties (L-202, Pate Blanc Mn 1, Stegaru 65, and Ligerito) with high globulin accumulation after 24 h imbibition compared with those of varieties (Bico Branco, Koshihikari, Lomello, and Okshitmayin) with low globulin under both conditions (Figure 4A,B and Figure 5A,B). Meanwhile, the H_2_O_2_ concentration in dry and germinating seeds was also measured. By comparison, significantly higher H_2_O_2_ contents were observed in varieties with high globulin accumulation compared with those varieties with low globulin content under both conditions (Figure 4C,D and Figure 5C,D). The mean relative H_2_O_2_ content of the varieties with high globulin accumulation to the varieties with low globulin accumulation was 1.85 and 2.81 in dry and germinating seeds under normal conditions, respectively, while the corresponding data were 2.30 and 1.90 under aged conditions. These results suggest that the higher accumulation of globulin contributes to ROS accumulation during seed germination in rice.

### 2.4. H_2_O_2_ Treatment Improves Seed Vigor

**Figure 6:**
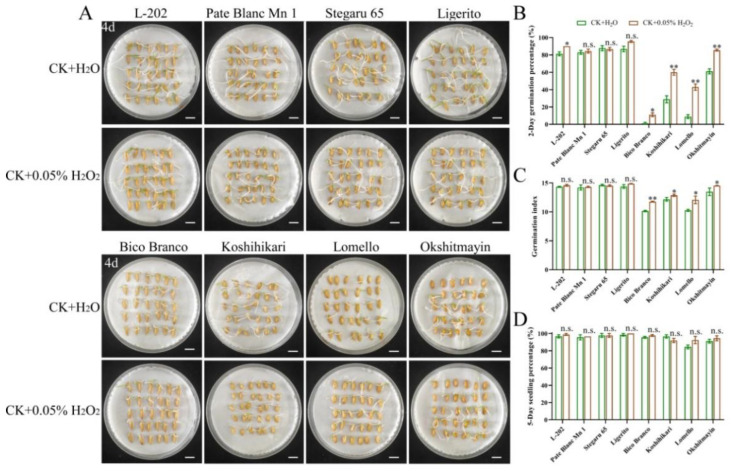
Effects of H_2_O_2_ treatments on seed vigor among varieties under normal conditions. (**A**) Representative images of seed germination in Petri dishes. Bar = 10 mm. (**B**) Two-day germination percentage; (**C**) Germination index; (**D**) Five-day seedling percentage. Each column represents the means ± SD, *n* = 3. * and ** indicate the significant difference compared to normal conditions at 5% and 1% levels, respectively, according to Student’s *t*-test. n.s. represents not significant.

**Figure 7:**
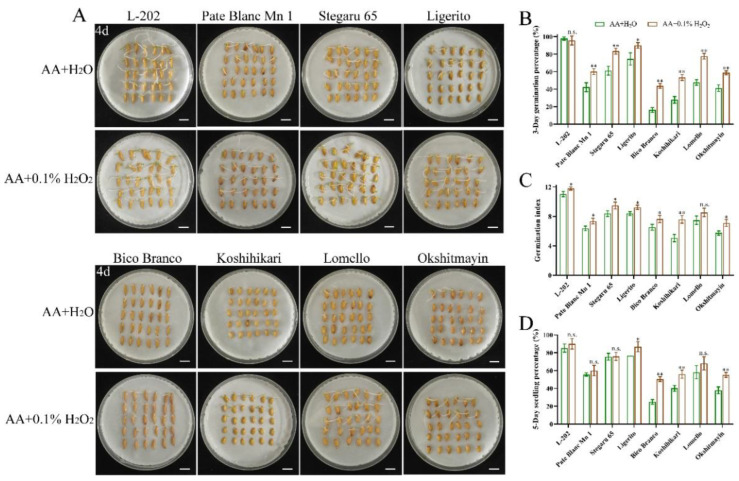
Effects of H_2_O_2_ treatments on seed vigor among varieties under aged conditions. (**A**) Representative images of the seed germination in Petri dishes. Bar = 10 mm. (**B**) Two-day germination percentage; (**C**) Germination index, (**D**) Five-day seedling percentage. AA means artificial aging. Each column represents the means ± SD, *n* = 3. * and ** indicate the significant difference compared to normal conditions at 5% and 1% levels, respectively, according to Student’s *t*-test. n.s. represents not significant.

According to the results above, we speculated that globulin affects seed vigor and might be involved in the ROS pathway. To confirm this hypothesis, the effects of exogenous H_2_O_2_ treatments on seed vigor were further analyzed under normal and aged conditions (Figure 6 and Figure 7). We observed that exogenous 0.05% H_2_O_2_ treatment significantly enhanced seed vigor, including germination percentage and germination index, in varieties (Bico Branco, Koshihikari, Lomello, and Okshitmayin) with low globulin accumulation compared to the control (H_2_O) under normal conditions (Figure 6A–C). However, no similar results were observed in varieties (L-202, Pate Blanc Mn 1, Stegaru 65, and Ligerito) with high globulin accumulation. Similarly, exogenous 0.1% H_2_O_2_ treatment significantly enhanced seed vigor, including germination percentage, germination index, and seedling percentage under aged conditions, especially in varieties with low globulin accumulation compared to the control (H_2_O) (Figure 7). It suggests that the insufficient ROS content in varieties with low globulin accumulation might cause its low seed vigor, and the low concentration of ROS as a positive signal contributes to seed vigor.

### 2.5. Evaluation of Seed Vigor in Commercial Varieties by Globulin Content Detection

**Figure 8:**
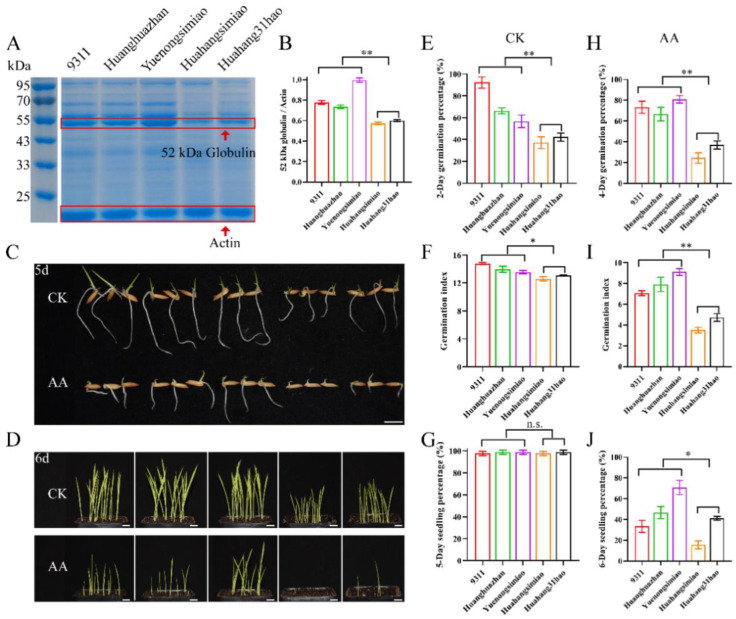
Evaluation of seed vigor using the detection of globulin content in popularly cultivated varieties. (**A**) 52 kDa globulin content. (**B**) The quantification of 52 kDa globulin accumulation by using ImageJ. (**C**) Representative images of the seed germination in Petri dishes under normal and aged conditions. (**D**) Images of seedling establishment by direct seeding in soils. Bar = 10 mm. (**E**) Two-day germination percentage, (**F**) Germination index, and (**G**) Five-day seedling percentage under normal conditions (CK). (**H**) Four-day germination percentage, (**I**) Germination index, and (**J**) Six-day seedling percentage under artificially aged conditions (AA). Each column represents the means ± SD, *n* = 3. * and ** indicate the significant difference compared to normal conditions at 5% and 1% levels, respectively, according to Student’s *t*-test. n.s. represents not significant.

As mentioned above, globulin accumulation is positively associated with seed vigor in rice. To clarify the application for the evaluation of seed vigor through globulin content detection, five commercial varieties (9311, Huanghuazhan, Yuenongsimiao, Huahangsimiao, and Huahang31hao) randomly selected from South China were used to analyze the relationship between globulin content and seed vigor. By comparison, a higher level of 52 kDa globulin was observed in 9311, Huanghuazhan, and Yuenongsimiao (Figure 8A), which also possessed higher seed vigor, including germination percentage, germination index, and seedling percentage, under normal and aged conditions (Figure 8B–I). Expectedly, Huahangsimiao and Huahang31hao varieties had a lower accumulation of 52 kDa globulin and lower seed vigor. The mean relative germination percentage, germination index, and seedling percentage of the varieties with high globulin accumulation to the varieties with low globulin accumulation were 1.63, 1.07, and 1.00 under normal conditions, respectively, while the corresponding data were 2.21, 2.01, and 1.77 under aged conditions. Taken together, the evaluation of seed vigor by detecting globulin content is feasible.

## 3. Discussion

Seed vigor is an important agronomic trait for direct seeding in rice. The accumulated SSPs will be turned into amino acids and organic nitrogen reserves for seed germination. Meanwhile, SSPs have been reported to have involvement in seed longevity in *Arabidopsis* [15]. We thus assumed that higher globulin accumulation contributes to seed vigor in rice. Rapid seed germination and high seed aging tolerance are vital indications of seed vigor. To confirm the above speculation, randomly selected rice varieties with high and low globulin accumulation were used to investigate the role of globulin on seed germination and seed aging tolerance in this study. Expectedly, our results suggest that the accumulation of globulin is positively associated with speed germination and seed aging tolerance in rice. Our previous study showed that the elite haplotype (Hap1) of the *OsCDP3.10* gene that regulates the synthesis of 52 kDa globulin is positively correlated with seed vigor [9]. Similar results were observed that the Hap1 varieties L-202, Pate Blanc Mn 1, and Ligerito have higher seed vigor compared with varieties Bico Branco, Koshihikari, Lomello, and Okshitmayin without Hap1 in this study. Moreover, the data of agronomic traits, such as plant height and heading date, of the varieties used here were compared according to public data (Rice Diversity, http://www.ricediversity.org/; accessed on 1 June 2022). No significant differences between plant height and heading date were observed between high-vigor varieties and low-vigor varieties. The globulin content might be a useful marker for the selection of favorable rice varieties with high seed vigor for direct seeding. Here, popularly cultivated varieties such as 9311, Huanghuazhan, and Yuenongsimiao in Southern China were selected for direct seeding due to their high globulin accumulation and seed vigor.

It has been reported that *OsCDP3.10* encoding 52 kDa globulin improves seed vigor by enhancing H_2_O_2_ accumulation during seed germination [9]. The high abundance of SSPs plays an important role in ROS-buffering during the dry storage of seeds [15]. In this study, we observed that the ROS levels were significantly increased in early germinating seeds of varieties with higher globulin content compared to those varieties with lower globulin content under normal and aged conditions. Meanwhile, seed vigor was significantly improved by H_2_O_2_ treatment in rice. Thus, we speculated that globulin accumulation will contribute to ROS accumulation in the early germinating seeds of rice to enhance seed vigor. However, for a long time, it was assumed that the higher ROS level is detrimental to seed viability during seed storage [16]. Here, the ROS accumulation in the early germinating seeds might be used as a cell signal to promote seed germination [17]. It is well known that hormones, such as abscisic acid (ABA), gibberellin (GA), and auxin, play vital roles in seed germination [18,19,20]. However, whether globulin improved seed vigor by influencing hormones during seed germination needs to be further investigated.

In summary, we validated the positive relationships between seed vigor and globulin accumulation in this study. The identification of globulin content might be a useful approach in selecting favorable varieties with high seed vigor for direct seeding. Seed vigor is established during seed development and maturation stages with protein storage. Whether globulin is involved in the regulation of seed development is still unclear. Previously, it has been reported that protein content is negatively correlated with grain chalkiness [21,22,23]. The negative correlation between globulin content and chalkiness was also observed in this study. However, the mechanisms of chalkiness regulated by globulin need to be further investigated in the future.

## 4. Materials and Methods

### 4.1. Plant Materials

The varieties L-202, Pate Blanc Mn 1, Stegaru 65, Ligerito, Bico Branco, Koshihikari, Lomello, and Okshitmayin, as well as 9311, Huanghuazhan, Yuenongsimiao, Huahangsimiao, and Huahang31hao from South China, were used for the following investigation. The plants were grown in an experimental field at South China Agricultural University (Guangzhou, Guangdong, China). All seeds were harvested at maturity, and then well-filled seeds were dried and randomly selected for experiments.

### 4.2. Evaluation of Seed Vigor

The germination assay was conducted according to He et al. [3] under normal and aged conditions. The aged seeds were obtained after the treatment of 58 ℃ water for 1 h and then dried at 35 ℃ for 24 h. Seeds were imbibed in 9 cm-diameter Petri dishes and were directly sowed in soils at 25 ± 1 °C conditions for 7 or 10 days, respectively. The germination standard is radicle (1 mm) breaking through testa, and seedlings are considered established when the root length reached seed length. Three biological replications were used.

### 4.3. Total Protein Content Assay

The grains were harvested 35 days after flowering (DAF) for the assay of total protein content, determined by a CBB colorimetry method [24]. Briefly, approximately 1 g of seeds were rapidly frozen and homogenized into a powder, and then 0.1 g of powder was added to a clean 2 mL collection tube with 1 mL of cold PBS buffer (PH = 7.0) at 4 °C. The mixture was put in an ice-bath for 20 min, and then centrifuged at 10,000× *g* for 10 min at 25 °C. The supernatant was transferred into a new 2 mL collection tube. The extraction step was repeated twice. The absorbance of the supernatant mixture was detected at 595 nm. Three biological replicates were used.

### 4.4. Globulin Content Assay

The globulin was extracted according to Peng et al. [9]. The molecular weight of the protein was detected by 10% sodium dodecyl sulfate polyacrylamide gel electrophoresis (SDS-PAGE). A solution (0.1% (*w*/*v*) CBB R-250, 45% (*v*/*v*) methanol, and 45% (*v*/*v*) glacial acetic acid) of Coomassie Brilliant Blue (CBB) was used for protein staining for 5 h, and then a destaining solution (1% methanol, 1% glacial acetic acid, and 8% distilled H_2_O) was used to wash the gels, and then photographed with a digital camera.

### 4.5. Evaluation of ROS Level

Nitroblue tetrazolium (NBT) staining and 3,3′-diaminobenzidine (DAB) staining was used to detect O_2_^.-^ and H_2_O_2_ content according to Liu et al. [25] and Hu et al. [26], respectively. The level of H_2_O_2_ was detected using the commercial assay kits (Beijing Solarbio Science & Technology Co., Ltd., Beijing, China). Three biological replicates were used.

### 4.6. Seed Treatments with H_2_O_2_

Seeds were imbibed in Petri dishes (diameter 9 cm) with 10 mL 0.05% or 0.1% H_2_O_2_ solution as the experimental groups, as well as 10 mL distilled H_2_O as a control, for 7 days at 25 °C. Seed vigor was assayed according to the above descriptions. Three biological replicates were used.

### 4.7. Data Analysis

Experimental data were analyzed using the SAS software (Cary, NC, USA), and significant differences among samples were compared using Student’s *t*-test.
